# Charge-Transfer Resonance and Electromagnetic Enhancement Synergistically Enabling MXenes with Excellent SERS Sensitivity for SARS-CoV-2 S Protein Detection

**DOI:** 10.1007/s40820-020-00565-4

**Published:** 2021-01-05

**Authors:** Yusi Peng, Chenglong Lin, Li Long, Tanemura Masaki, Mao Tang, Lili Yang, Jianjun Liu, Zhengren Huang, Zhiyuan Li, Xiaoying Luo, John R. Lombardi, Yong Yang

**Affiliations:** 1 State Key Laboratory of High-Performance Ceramics and Superfine Microstructures, Shanghai Institute of Ceramics, Chinese Academy of Sciences, 1295 Dingxi Road, Shanghai, 200050 People’s Republic of China; 2 Graduate School of the Chinese Academy of Sciences, No.19(A) Yuquan Road, Beijing, 100049 People’s Republic of China; 3 Center of Materials Science and Optoelectronics Engineering, University of Chinese Academy of Sciences, Beijing, 100049 People’s Republic of China; 4 School of Physics and Optoelectronics, South China University of Technology, Guangzhou, 510641 People’s Republic of China; 5 Department of Frontier Materials, Nagoya Institute of Technology, Nagoya, 466-8555 Japan; 6 State Key Laboratory of Oncogenes and Related Genes, Shanghai Cancer Institute, Renji Hospital, Shanghai Jiaotong University School of Medicine, Shanghai, 200032 People’s Republic of China; 7 City College, CUNY, New York, NY USA

**Keywords:** Nb_2_C and Ta_2_C MXenes, SERS sensitivity, PICT resonance, SARS-CoV-2 S protein

## Abstract

The outbreak of coronavirus disease 2019 has seriously threatened human health. Rapidly and sensitively detecting SARS-CoV-2 viruses can help control the spread of viruses. However, it is an arduous challenge to apply semiconductor-based substrates for virus SERS detection due to their poor sensitivity. Therefore, it is worthwhile to search novel semiconductor-based substrates with excellent SERS sensitivity. Herein we report, for the first time, Nb_2_C and Ta_2_C MXenes exhibit a remarkable SERS enhancement, which is synergistically enabled by the charge transfer resonance enhancement and electromagnetic enhancement. Their SERS sensitivity is optimized to 3.0 × 10^6^ and 1.4 × 10^6^ under the optimal resonance excitation wavelength of 532 nm. Additionally, remarkable SERS sensitivity endows Ta_2_C MXenes with capability to sensitively detect and accurately identify the SARS-CoV-2 spike protein. Moreover, its detection limit is as low as 5 × 10^−9^ M, which is beneficial to achieve real-time monitoring and early warning of novel coronavirus. This research not only provides helpful theoretical guidance for exploring other novel SERS-active semiconductor-based materials but also provides a potential candidate for the practical applications of SERS technology.

**Electronic supplementary material:**

The online version of this article (10.1007/s40820-020-00565-4) contains supplementary material, which is available to authorized users.

## Highlights

- Nb_2_C and Ta_2_C MXenes exhibit remarkable SERS performance with the enhancement factors of 3.0 × 10^6^ and 1.4 × 10^6^, which is synergistically enabled by the PICT resonance enhancement and electromagnetic enhancement.
- The excellent SERS sensitivity endows Ta_2_C MXene with the capability to sensitively detect and accurately identify the SARS-CoV-2 spike protein, which is beneficial to achieve real-time monitoring and early warning of novel coronavirus.

**Electronic supplementary material:**

The online version of this article (10.1007/s40820-020-00565-4) contains supplementary material, which is available to authorized users.

## Graphical abstract

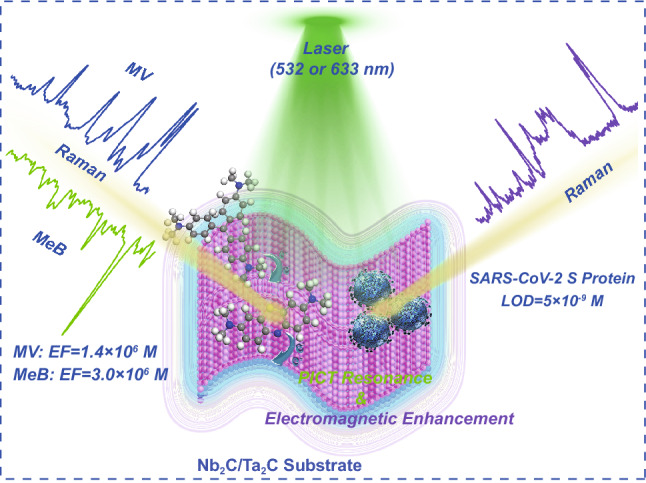

## Introduction

Surface-enhanced raman scattering (SERS), as a powerful spectral detection technology, has attracted a significant interest for chemical and biological analysis. The SERS technique is expected to develop into a practical technology due to its characteristics of high sensitivity, high accuracy, non-destructive, trace detection, and the real-time and in situ rapid detection, especially in the promising applications of toxin or virus detection [1], environmental science [2], and biosensor [3–5]. All along, viral infections have seriously threatened the human health, especially the outbreak of coronavirus-disease-2019 (COVID-19) caused by novel coronavirus in December 2019. It is a kind of severe acute respiratory syndrome-coronavirus-2 (SARS-CoV-2) and has been declared a public health emergency of international concern due to its highly contagious. Recent research indicated that SARS-CoV-2 has been detected in contaminated water, and pointed out that the detection of virus in contaminated water is expected to reveal the true scale of coronavirus-infected person, thus to effectively control the spread of SARS-CoV-2 [6]. Therefore, it is critical to sensitively detect and accurately identify SARS-CoV-2 in patients’ body fluids or contaminated water to complete the real-time monitoring and early warning of viruses.

At present, noble metal nanoparticles are frequently used as effective SERS substrates for virus detection due to their ultra-high SERS sensitivity, which are mainly enhanced by the electromagnetic mechanism (EM) [7–9]. Nevertheless, the poor biocompatibility and the ability to denature proteins are two culprits that limit the practical applications of noble metal substrates in virus detection. Compared to noble metal substrates, semiconductor materials exhibit many charming advantages of biocompatibility, high spectral stability, strong anti-interference ability and selective SERS enhancement of target molecules, which make semiconductor-based SERS substrates being widely applied in the identification and sensing of biomolecules. Moreover, it is encouraging that more and more research has been engaging in exploiting novel semiconductor-based SERS substrates [10–25], where the chemical mechanism (CM) played a dominant role [26, 27]. However, the semiconductor-based SERS technique as a new analytical methodology has not been developed into a practical technology as far as initially predicted. The major obstacle in semiconductor-based SERS substrates is their relatively weak SERS enhancement factors, which is difficult to meet the requirement of practical detection. Therefore, a task of top priority is to explore novel semiconductor-based substrates with excellent SERS performance and optimized the SERS sensitivity by an effective experimental design.

Motivated by the research and development of various two-dimensional materials, MXenes have attracted increasing attentions [28, 29]. As an atomically thin two-dimensional material, MXene is a kind of semi-metal material and exhibits abundant intriguing properties of the metallic conductivity, hydrophilicity, high light transmission, biocompatibility, tunable electronic structure, high carrier mobility, and enable to realize strong light-matter interactions at mid-IR and THz frequencies [30–32]. On the other hand, MXene also exhibits a flat surface with large specific surface area, whose coordination number of surface atoms is seriously insufficient. The stronger photo-induced charge transfer (PICT) resonance benefited from 2D structure than the corresponding 3D structure is generated due to the easier combination of surface atoms with adsorbed molecules [14, 33–35]. In a word, above-mentioned properties make MXenes more likely to exhibit excellent SERS sensitivity. Recent research has reported that only Ti_3_C_2_ and Ti_2_N MXene exhibited SERS sensitivity and challenges also remained regarding the improvement of SERS sensitivity and the practical applications of MXenes [35–38]. As research in the SERS activity of Ti_3_C_2_ and Ti_2_N MXene has broadened, we drive focus toward the other MXene materials. Studies on the SERS performance of Nb_2_O_5_ nanoparticles and Ta_2_O_5_ nanorods indicated that the semiconductor nanoparticles with five-deputy cations showed a better SERS performance comparing with other oxide semiconductor substrates [10, 11]. In addition to the factors of the doping and morphology regulation, multi-coordination cations of Nb^5+^ and Ta^5+^ are another reason that cannot be ignored. Because the more excited electrons are existed outside the nucleus of Nb^5+^ and Ta^5+^, which is not only conducive to the charge transfer between semiconductor and molecule but also beneficial to the generation of vacancy defects. Therefore, combining the above-mentioned advantages of MXenes and Nb, Ta multi-coordination cations in the SERS filed, Nb_2_C and Ta_2_C MXene materials grab our attention, which are expected to exhibit excellent SERS performance.

**Scheme 1:**
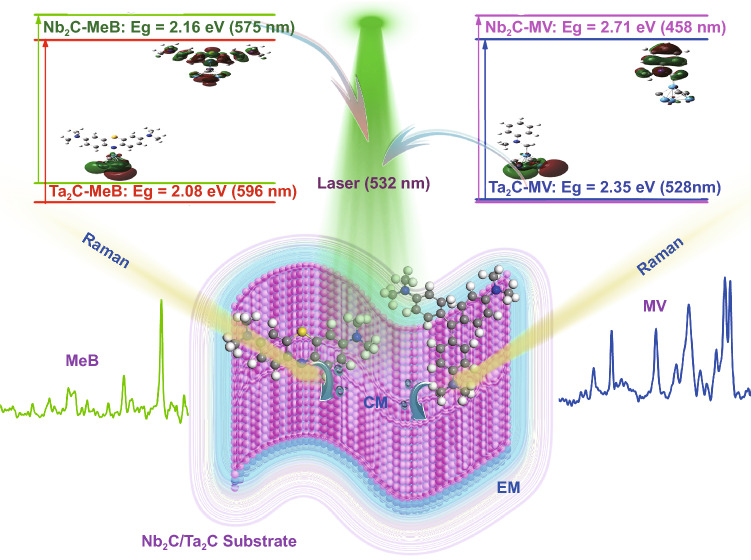
Mechanism diagram of the experimental design for optimizing SERS sensitivity of substrates

Inspired by the desire for more sensitive semiconductor-based SERS substrates, the contribution of charge transfer between semiconductor-based substrate and probe molecule to SERS activity has been comprehensively researched [39, 40]. Therefore, considering matching semiconductor and molecule energy levels to achieve PICT resonance may be an effective strategy to optimize SERS sensitivity. In respect of this, we presented an effective experimental design to optimize the SERS sensitivity (Scheme 1). The essence of this experimental design is matching the energy of excitation laser with the HOMO–LUMO (the highest unoccupied molecular orbitals, the lowest occupied molecular orbitals) energy levels of the molecule-cluster complex to realize the PICT resonance [36, 41, 42]. Firstly, density functional theory (DFT) calculations were performed to characterize the contribution of CM to the SERS activity, as well as to select the appropriate probe molecules and to determine the optimal resonance excitation wavelength of laser. Then, based on the finite difference time domain (FDTD) method, the surface electromagnetic field distribution was calculated to represent the contribution of EM to SERS activity. Finally, with the guidance of calculated results, the experimental research on the SERS performance of Nb_2_C and Ta_2_C MXene materials were carried out.

In this work, motivated by the require of virus detection for ultra-sensitivity semiconductor-based SERS substrates, both Nb_2_C and Ta_2_C MXenes were theoretically predicted to exhibit a remarkable SERS sensitivity due to the contribution of CM and EM, and the optimal resonance excitation wavelengths of Nb_2_C-MeB (methylene blue) complex and Ta_2_C-MV (methyl violet) complex were both determined to 532 nm by concerning the PICT resonance. Then, with the guidance of theoretical results, Nb_2_C and Ta_2_C MXene substrates with excellent SERS performance were originally reported and the SERS enhancement factors (detection limits) of Nb_2_C-MeB complex and Ta_2_C-MV complex are successfully optimized to reach up to 3.0 × 10^6^ (10^−8^ M) and 1.4 × 10^6^ (10^−7^ M), respectively. Moreover, the sensitive detection of SARS-CoV-2 S protein and the accurate identification of Raman peaks have been completed by using Ta_2_C MXene substrate, and its detection limit is down to 5 × 10^−9^ M, which can conduce to control the spread of SARS-CoV-2 virus based on SERS technology.

## Experimental Section

### First-Principles Calculation

The first-principles calculations based on density functional theory (DFT) [43] are employed to investigate the chemical enhancement mechanism of Nb_2_C and Ta_2_C MXene materials in SERS field. All the calculations are accomplished by the CASTEP and Gauss09 program [44]. Firstly, the supercell expansion with 2 × 2 × 1 of Nb_2_C and Ta_2_C MXene crystal structures was built. After geometry optimization, the electronic structure, absorption, and dielectric function spectra were calculated by the CASTEP program. During the calculation process, the PBE method in the generalized gradient approximation (GGA) was adopted to describe the periodic boundary conditions and the inter-electronic exchange–correlation energy [45]. The interaction potential between ion core and valence electrons was achieved by the ultra-soft potential (Ultrasoft). The cut-off energy of plane wave was chosen as 480 eV in the wave vector K-space. And the Brillouin zone of 3 × 3 × 1 was summed according to the special K-point of Monkhorst–Park [46]. The calculation accuracy of the crystal structure system reaching the convergence state is set as follows: the total energy change of the system stable within 10^−5^ eV, the force acting on each atom in the unit cell less than 0.03 eV Å^−1^, the residual stress of the unit cell and the tolerance deviation within 0.05 GPa and 10^−3^ Å, respectively. During the geometric optimization process, the weak interaction was considered and the added U value for the Nb and Ta atom are 3.0. Then, 4-MBA, MeB, MV molecule models and Phe, His, Trp, Tyr amino acid molecule models, Nb_2_C and Ta_2_C cluster models, as well as their complex models were built. The ground state geometric optimization, static Raman spectra, charge difference distribution, and the molecular orbital were calculated by the Gauss09 program. The Becke’s three-parameter hybrid exchange functional and Lee, Yang, and Parr’s (B3LYP) exchange functional combined with basis sets are adopted for all calculations. To ensure keeping all structures in a stable state with the lowest energy, the probe molecules and cluster models were optimized without virtual frequencies. The 6-311+G (*d*, *p*) group including a polarization function and a diffusion function was selected for the C, H, O, N, F, and S atoms in the probe molecules system. The transition metal Nb, Ta atoms were described by the Lanl2dz basic group. It has been verified that both the B3LYP/6-311+G(*d*,*p*) and the B3LYP/Lanl2dz basic group level were sufficient to describe the interaction between organic molecules and transition metal compound clusters.

### Finite-Difference Time-Domain Simulations

The electric field distributions of MXene nanosheets were calculated by the finite difference time domain (FDTD) solution, a numerical technique to solve boundary-value problem. This method provides a convenient and general approach for calculating the optical response of nanostructures of arbitrary geometry to an incident light wave. In the Raman scattering process, the enhancing factor of Raman radiated power is shown in below:

1 $$G = \frac{{\left| {I\left( {r_{\infty } ,\omega_{R} } \right)} \right|}}{{\left| {I_{vac} \left( {r_{\infty } ,\omega_{R} } \right)} \right|}} = \frac{{\left| {E\left( {r_{\infty } ,\omega_{R} } \right)} \right|^{2} }}{{\left| {E_{vac} \left( {r_{\infty } ,\omega_{R} } \right)} \right|^{2} }} \approx \left[ {f_{1} \left( \omega \right)} \right]^{2} \times f_{2} \left( {\omega_{R} } \right)^{2}$$

Due to the little difference is existed between the Raman radiation frequency and the incident frequency, the local light field enhancing factor $f_{1} \left( \omega \right)$ caused by surface plasmon is approximately equal to the radiation light field enhancing factor $f_{2} \left( \omega \right)$.

2 $$f_{1} \left( \omega \right) = \frac{{\left| {E\left( {r_{0} ,\omega } \right)} \right|}}{{\left| {E_{0} \left( {r_{0} ,\omega } \right)} \right|}} \approx \frac{{\left| {E\left( {r_{\infty } ,\omega_{R} } \right)} \right|}}{{\left| {E_{0} \left( {r_{\infty } ,\omega_{R} } \right)} \right|}} = f_{2} \left( {\omega_{R} } \right)$$

Therefore, the SERS enhancing factor is approximately equal to the fourth power of enhancing factor for local field.

3 $$G\left( {r_{0} } \right) = \frac{{\left| {E\left( {r_{0} ,\omega } \right)} \right|^{4} }}{{\left| {E_{0} \left( {r_{0} ,\omega } \right)} \right|^{4} }} = \frac{{\left| E \right|^{4} }}{{\left| {E_{0} } \right|^{4} }}$$

Among them, $E_{0}$ is the light electric amplitude and usually chosen to 1 V m^−1^, and $E$ can be calculated by FDTD solution. Therefore, the SERS enhancing factor $\left| E \right|^{4} /\left| {E_{0} } \right|^{4}$. contributed by EM can be obtained by FDTD calculation. MXene geometric model was constructed as follows: thirty-five layers MXene with a diameter of 1 μm, thickness of 0.25 nm, and inter-layer distance of 0.28 nm. Each layer of MXene (Ti_3_C_2_, Nb_2_C, Ta_2_C) material was set as a 2D conductor and the electrical conductivity set as 0.5 × 10^6^, 1.33 × 10^6^, and 2.59 × 10^6^ $\Omega ^{ - 1} {\text{m}}^{ - 1}$ [47]. We aligned the TFSF source (532 nm, x polarization) parallel to the normal to the MXene nanosheets. To obtain accurate results in the simulations, the Yee cell size was set to 2 × 2 × 0.1 nm^3^, and external medium of vacuum was used.

### Sample Preparation

The Nb_2_C and Ta_2_C MXene nanosheets were synthesized successfully by a two-step exfoliation process according to the literature [48]. Firstly, HF acid etching method was adopted to completely remove Al atoms of NbAl_2_C and TaAl_2_C MAX phase. The 3.0 g of NbAl_2_C and 4.0 g of TaAl_2_C MAX powder, which are purchased from Forsman Scientific of Beijing, were immersed in 30 mL of a 40% HF aqueous solution (Aladdin Co., Ltd., Shanghai, China). At room temperature, the NbAl_2_C and TaAl_2_C mixed solutions were electromagnetic stirred 15 days and 2 days, respectively. Then, the etched Nb_2_C and Ta_2_C MXene powder were centrifugalized and washed 5 times with water and ethanol. Then, in order to enlarge the interlayer distance of stacked nanosheets, the prepared Nb_2_C and Ta_2_C MXene powder dispersed in 50 mL of tetrapropylammonium hydroxide (TPAOH) (25 wt% aqueous solution, Macklin Co., Ltd., Shanghai, China) and electromagnetic stirred 3 days or longer time at room temperature to obtain the more uniform and thinner nanosheets. These delaminated Nb_2_C and Ta_2_C powder were collected by centrifugation and washed more than three times with ethanol to remove the residual TPAOH. Finally, the synthesized sample powder was dispersed in deionized water and freeze-dried to improve the stratification effect.

### Characterizations

The micro-morphology of Nb_2_C and Ta_2_C MXene was determined by FEI Magellan 400 field emission scanning electron microscopy (FESEM). The transmission electron microscopy (TEM), high-resolution TEM (HRTEM), energy-dispersive X-ray spectroscopy (EDS), and selected area electron diffraction (SAED) images were performed on a JEM-2100F field emission source transmission electron microscope (200 kV). The powder X-ray diffraction (XRD) measurements were made using a Rigaku D/MAX-2200 PC XRD system (parameters: Cu Kα radiation, *λ* = 1.54 Å, 40 mA and 40 kV). And the Thermo Fisher Scientific ESCAlab250 provided the X-ray photoelectron spectroscopy (XPS). Atomic force microscope (AFM) images were measured by a Veeco DI Nanoscope Multi Mode V system.

### SERS Measurements

Three probe molecules of 4-MBA, MV, MeB aqueous solution with the different concentration of 10^−5^–10^−8^ M were used to investigate the SERS performance of Nb_2_C and Ta_2_C MXene nanosheets. To explore the SERS detection capability of Nb_2_C and Ta_2_C MXene substrates for virus, Raman spectra of the 5 × 10^−9^ M SARS-CoV-2 S protein were detected. For each test, the 0.02 g of synthesized sample powder was immersed in 30 mL of molecule aqueous solution and treated with ultrasound for 4 h. A dose of mixture solution with a volume of 1 μL was dropped on the surface of glass substrate and dried at room temperature. All the Raman spectra of dye molecules were obtained by Renishaw inVia Reflex Raman spectrometer with the laser power of 0.5 mW at 532 nm and the accumulation time was 20 s. As for SARS-CoV-2 S protein, the excitation intensity used was 17 mW × 1% at 633 nm, and the laser beam was focused to a spot about 1 μm in diameter with a 50× microscope objective and the accumulation time of 5 s. Three different points on each substrate were tested and selected the strongest intensity of the Raman spectrum to calculate the SERS EF value. It is worth noting that the Raman intensity of carbide substrates should be subtracted from all Raman spectra of substrate-molecule complexes at their corresponding excitation wavelength. The reason can be ascribed to the Raman peaks at 1610 cm^−1^ representing C–C vibration mode are found in the Raman spectra of Nb_2_C or Ta_2_C substrates except for the fluorescence peak of Ta_2_C substrates near 1350 cm^−1^ under the excitation of 633 nm laser, which is coincident with the Raman shift of main peaks in probe molecules (Fig. S1).

## Results and Discussion

### Theoretical Prediction of SERS Activity

Inspired by immense technological promise, Nb_2_C and Ta_2_C MXene materials have attracted significant interest for the applications in the photothermal tumor eradication, supercapacitor, sensor and photocatalyst [48–51]. However, the potential applications of Nb_2_C and Ta_2_C MXene in the SERS field have never been reported. In this work, the first-principles calculations based on DFT and FDTD simulations were performed to give a comprehensive and clear prediction of SERS performance about Nb_2_C and Ta_2_C MXene substrates.

The partial density of states (PDOS), absorption and dielectric function spectra of Nb_2_C and Ta_2_C bulk structures were calculated by the CASTEP package. Studies on the PDOS (Fig. S2a) indicate that Nb_2_C and Ta_2_C MXenes are semi-metal-like properties. Both Nb_2_C and Ta_2_C MXenes have higher electron state densities near the Fermi energy level, which are mainly contributed by the *d* orbital electrons of Nb and Ta. Generally, the higher electron state densities of substrate materials can generate a large amount of charge transfer and effectively improve the vibronic coupling of several resonances in the substrate–molecule system, resulting in the high-efficiency PICT resonance [14]. Therefore, Nb_2_C and Ta_2_C MXenes as SERS substrates are expected to exhibit excellent SERS activity. Additionally, the ultraviolet–visible absorption spectra (Fig. S2b) show that Nb_2_C and Ta_2_C MXenes exhibit a strong optical absorption in the visible region, which indicate that the outer electrons of these two MXenes are more easily excited by the visible light. As we all know, if the imaginary part of a material’s dielectric constant is greater than zero and the real part of the dielectric constant is less than zero in all wavelengths, it would be an ideal plasmonic candidate [52]. According to the dielectric function spectra (Fig. S2b), the wavelength region with a negative real part of dielectric constant spans the entire visible region. Therefore, Nb_2_C and Ta_2_C MXenes as SERS substrate are quite likely to show the excellent plasmonic property. Based on above calculations of intrinsic properties, Nb_2_C and Ta_2_C MXenes are likely to exhibit an excellent Raman enhancement in both CM and EM.

Recent research has demonstrated that CM governs the molecular and vibrational selectivity of SERS and the mechanism of the 2D material-based Raman enhancement effect is mainly believed to the CM [53]. Herein, the static Raman spectra, the charge difference distribution and the molecular orbital, were calculated to characterize the contribution of the CM to SERS activity. Firstly, three kinds of probe molecules 4-MBA (4-Mercaptobenzoic acid), MeB, MV with a strong optical absorption peak near the excitation wavelength of 270, 580, and 660 nm were selected (Fig. S3b) to illustrate the influence of charge transfer resonance on SERS activity. 4-MBA, MeB, MV molecules are bonded to Nb_2_C (Ta_2_C) cluster via the Nb-S (Ta-S) bond, Nb-N (Ta-N) bond, Nb-C (Ta-C) bond, respectively. The calculated and experimental Raman spectra of 4-MBA, MeB, MV molecules showed that all Raman peaks of theoretical calculations are quite consistent with our experimental results, which indicates the DFT calculation method is accurate enough to describe these systems.

**Fig. 1:**
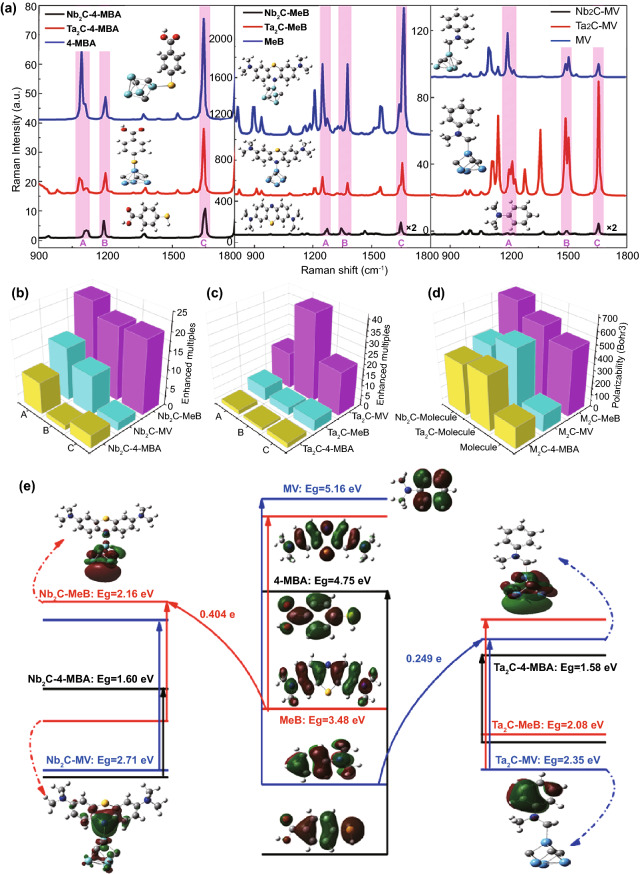
**a** Static Raman spectra of isolated molecules 4-MBA, MeB, MV, and complexes Nb_2_C-4-MBA, Ta_2_C-4-MBA, Nb_2_C-MeB, Ta_2_C-MeB, Nb_2_C-MV, Ta_2_C-MV. **b**, **c** The comparison of Raman enhanced multiples for Nb_2_C-based complexes (**b**): Nb_2_C-4-MBA, Nb_2_C-MeB, Nb_2_C-MV, and Ta_2_C-based complexes (**c)**: Ta_2_C-4-MBA, Ta_2_C-MeB, Ta_2_C-MV for calculated Raman models at A, B, C Raman shifts, respectively. **d** Calculated polarizabilities of isolated molecules: 4-MBA, MeB, MV, and these molecules adsorbed on Nb_2_C and Ta_2_C clusters, respectively. **e** Energy level distributions and HOMO/LUMO illustrations of 4-MBA, MeB, MV molecules, Nb_2_C-4-MBA, Nb_2_C-MeB, Nb_2_C-MV, Ta_2_C-4-MBA, Ta_2_C-MeB, Ta_2_C-MV complexes

Figure 1a shows the static Raman spectra of isolated molecules 4-MBA, MeB, MV, and those molecules adsorbed on Nb_2_C and Ta_2_C cluster. Obviously, these three molecules are all enhanced when adsorbed on either Nb_2_C or Ta_2_C cluster. The only difference is that probe molecules with different excitation wavelengths display different Raman enhancements on Nb_2_C and Ta_2_C MXene substrates. Examining the Raman intensity of MXene-molecule complexes (Fig. 1b, c), it shows that Nb_2_C has a better Raman enhancement on MeB molecule, and the SERS enhancement effect of the Ta_2_C-MV complex is more obvious. Additionally, the analysis of the polarizability (Fig. 1d) indicated that Nb_2_C and Ta_2_C MXenes can provide MeB and MV with a stronger amplification of the molecular polarization, respectively, thus resulting in the more significant SERS enhancement [54, 55]. Above results indicate that both Nb_2_C and Ta_2_C MXenes can serve as potential candidates for SERS active substrates. Previous research showed that the molecule–substrate complex would achieve the strongest Raman enhancement once the excitation frequency resonated with PICT. Therefore, the charge difference distribution and the molecular orbital were calculated to illustrate the higher SERS activity of Nb_2_C-MeB complex and Ta_2_C-MV complex (Fig. 1e). The adsorption of molecules on Nb_2_C and Ta_2_C clusters could form chemical bonds of the transition metal atom and nonmetal atom, which can serve as charge transfer channels to facilitate the redistribution of the electron cloud around molecules and MXene clusters. The electron rearrangement of LUMO and HOMO results in reducing the bandgap of the MXene-molecule complex, which is closer to their wavelength of radiated laser. Furthermore, it is possible for charge transfer to resonate with the frequency of excitation light, thus generating a more efficient PICT. The occupied and unoccupied orbitals of molecule-cluster complex were localized analysis based on Multiwfn program [56]. The accumulated region for electron density on HOMO is mainly localized on Nb-N bonding orbital of Nb_2_C-MeB complex and π bonding orbital on benzene ring of Ta_2_C-MV complex. And the depleted region for electron density on LUMO spans the whole Nb_2_C (Ta_2_C) cluster. Above results suggest that electrons more easily transfer from the HOMO orbital of MeB (MV) molecule to the conduction band of Nb_2_C (Ta_2_C) cluster. Moreover, it is found that the charge transfer quantity of Nb_2_C-MV complex (0.404 e) is larger than that of Ta_2_C-MV complex (0.249 e) due to due to the charge transfer distance of MeB molecule to Nb_2_C cluster is smaller. As for the isolated molecules MeB and MV, the HOMO–LUMO energy gap are 3.48 and 5.16 eV, which are too large to generate the charge transfer resonance by visible light. Whereas their HOMO–LUMO energy gap is significantly reduced to 2.16 and 2.35 eV when molecules absorbing on the Nb_2_C and Ta_2_C cluster. Obviously, the corresponding resonance excitation wavelengths of charge transfer process for MeB-Nb_2_C complex (575 nm) and MV-Ta_2_C complex (528 nm) are closer to the excitation wavelength of 532 nm, which is qualitatively consistent with the experimental ultraviolet–visible absorption spectral results (Fig. S4). Therefore, the optimal resonance excitation wavelengths of Nb_2_C-MeB complex and Ta_2_C-MV complex are both determined as 532 nm. The excitation wavelength of MeB (MV) molecule is tuned from ultraviolet region (less than 380 nm) to visible region (380–780 nm), which indicates that the more, new possible charge transfer processes are excited at the lower-energy level, thus realizing the PICT resonance at a specific laser wavelength.

It is worthwhile to note that the PICT from substrates to the probe molecules could increase the molecular electron density and result in an obvious change in the molecular polarization tensor, thus affecting the Raman intensity. Therefore, the polarizability could be used to evaluate the SERS activity of substrate materials. As shown in Fig. 1d, the calculated static polarizability of the Ta_2_C-MV (Nb_2_C-MeB) complex is larger than that of the Nb_2_C-MV (Ta_2_C-MeB) complex and MV (MeB) molecule, which indicates the stronger PICT and the more obvious Raman enhancement of Ta_2_C-MV and Nb_2_C-MeB. These conclusions are consistent with the static Raman spectra, and correspond to a fact that the charge transfer resonance excitation wavelength of Ta_2_C-MV (Nb_2_C-MeB) complex is closer to the excitation wavelength of 532 nm than the other complexes. In a word, both Nb_2_C and Ta_2_C MXene substrates are likely to exhibit an excellent SERS activity due to the contribution of PICT resonance enhancement at the optimal resonance excitation wavelength of 532 nm.

**Fig. 2:**
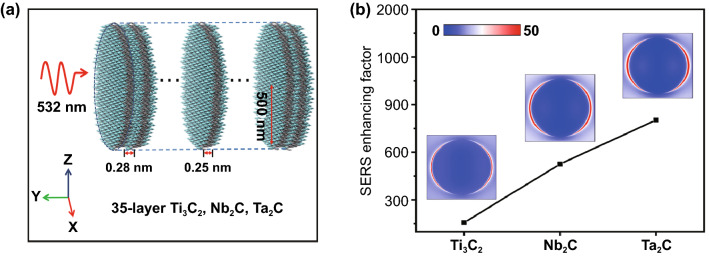
**a** Model of MXene (Ti_3_C_2_, Nb_2_C, Ta_2_C) for E-field intensity distribution simulation. **b** Simulated electric field intensity distribution of MXene (Ti_3_C_2_, Nb_2_C, and Ta_2_C) and corresponding SERS enhancing factor. Color bar represents electric field intensity

Based on the FDTD solution, the electric field distributions were calculated to characterize the contribution of the EM to SERS activity. Firstly, the circular stacked nanosheets model with a diameter of 1 μm was constructed and the incident light with a wavelength of 532 nm propagated along x axis (Fig. 2a). Moreover, the reported Ti_3_C_2_ MXene material with SERS activity was selected as a reference to compare the SERS sensitivity of Nb_2_C and Ta_2_C MXene. Figure 2b presents the electric field distributions of Ti_3_C_2_, Nb_2_C, and Ta_2_C MXene stacked nanosheets and SERS enhancing factors ($\left| E \right|^{4} /\left| {E_{0} } \right|^{4}$). The intensely enhanced electric fields are observed at the edge of the circular stacked nanosheets and their strongest electric field enhancement factors in xy cross section exist the following relation: Ti_3_C_2_ < Nb_2_C < Ta_2_C. The electromagnetic field of Ti_3_C_2_, Nb_2_C and Ta_2_C MXene can even reach up to 150, 525, 800 times of SERS enhancement, respectively. Exciting the dipole resonance of the MXene surface results in enhanced electromagnetic fields. Therefore, in the SERS activity contributed by EM, it is reasonable that Nb_2_C and Ta_2_C MXene materials are expected to show a significantly higher Raman enhanced effect than Ti_3_C_2_ MXene.

Based on the above research, a deep and comprehensive prediction of the SERS performance from Nb_2_C and Ta_2_C MXenes and their contributing mechanisms of EM and CM can be predicted, which provides the theoretical guidance for the following experimental research. Moreover, an efficient strategy is presented to realize an obvious improvement of SERS enhanced factor via confirming the optimal resonance excitation wavelength of 532 nm and the appropriate molecule.

### Characterization of MXenes Nanosheets

**Fig. 3:**
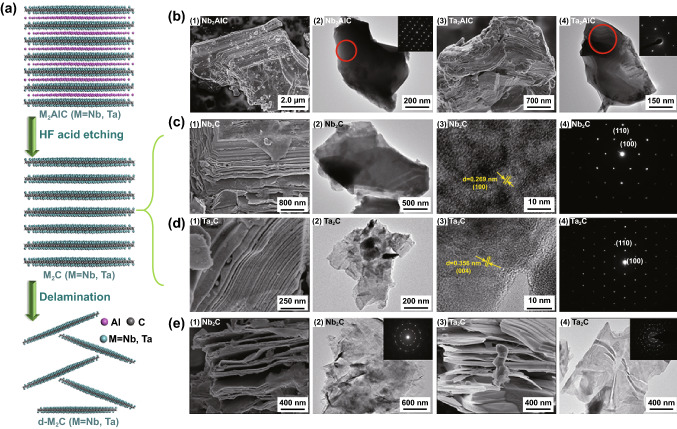
**a** Schematic illustration of the synthesis process for Nb_2_C and Ta_2_C nanosheets by the two-step exfoliation process. **b** SEM images of Nb_2_AlC (1) and Ta_2_AlC (3) bulk structure, TEM images and the corresponding SAED patterns (inset in Fig. 2, 4) of Nb_2_AlC (2) and Ta_2_AlC (4) bulk structure. **c**, **d** SEM images (1), TEM images (2), HRTEM images (3) and the corresponding SAED patterns (4) of Nb_2_C (**c**) and Ta_2_C (**d**). **e** SEM images of delaminated d-Nb_2_C (1) and d-Ta_2_C (3), TEM images and the corresponding SAED patterns (inset in Fig. 2, 4) of delaminated d-Nb_2_C (2) and d-Ta_2_C (4)

In this work, to ensure the P63/mmc space group of Nb_2_C and Ta_2_C MXenes, the HF etching of Al atoms and tetra propylammonium hydroxide (TPAOH) embedded stripping method were adopted to realize the synthesis of 2D MXene nanosheets. The schematic illustration of the synthetic process is shown in Fig. 3a. SEM, TEM, and HRTEM were used to study the morphological evolution of Nb_2_C and Ta_2_C MXenes. The Nb_2_AlC and Ta_2_AlC MAX phase exhibits a special morphology of layered ternary compound with a high-degree crystallinity of hexagonal system (Fig. 3b). Figure 3c, d shows the morphology and the selected area electron diffraction (SAED) of Nb_2_C and Ta_2_C MXenes after HF acid etching. SEM images show the typical layered structures of Nb_2_C and Ta_2_C MXenes, where several of the exfoliated nanosheets are stacked into bulks. Moreover, TEM images also demonstrate the formation of the layered structure, albeit of a rather large thickness. HRTEM images of the multilayered Nb_2_C and Ta_2_C MXenes show the clearly crystalline lattice, which correspond to the (100) and (004) planes of hexagonal structures with an inter-planer spacing of 0.269 and 0.356 nm, respectively. Moreover, SAED images of the multilayered Nb_2_C and Ta_2_C MXenes also confirm that the basal plane hexagonal symmetry structure and the high-degree crystallinity of the parent MAX phases have been preserved after HF treatment. In order to promote the charge transfer and increase the adsorption amount of probe molecules, the TPAOH chemical stripping method was adopted to increase the specific surface area of MXene nanosheets. SEM images of Nb_2_C and Ta_2_C show the layered morphology with significantly enlarged interlaminar distance after TPAOH chemical stripping. Moreover, compared with the contrast images before stripping, TEM images also show the electron-transparent flake structure and the thinner thickness of nanosheets, which can also be proved by the TEM images of the longitudinal section before stripping and atomic force microscopy (AFM) images after stripping (Figs. S5 and S6). Figure S5 shows that the thickness of Nb_2_C and Ta_2_C MXene are above 40 and 100 nm, respectively. The corresponding interlaminar distances are 0.913 and 1.8 nm, which are equivalent to the thickness of 2 atomic layers of Nb_2_C MXene and 3 atomic layers of Ta_2_C MXene. After the chemical exfoliation, a well-stacked thinner nanosheeting structures are revealed by AFM (Fig. S6). The stacked-layer thickness of delamination Nb_2_C and Ta_2_C MXenes are reduced to 20 and 40 nm. In addition, different from the SAED images before TPAOH chemical stripping, the SAED images after stripping tend to form polycrystalline diffraction rings due to the stacking of nanosheets with different orientations.

XRD patterns of Nb_2_AlC MAX and Nb_2_C MXene, Ta_2_AlC MAX, and Ta_2_C MXene are shown in Fig. S7a, d. The (002) peaks of delaminated Nb_2_C and Ta_2_C MXenes downshift toward a lower 2 $\theta$ angle of 4.328 and 7.472, respectively. The newly emerged low-angle (002) peaks of Nb_2_C and Ta_2_C MXenes indicate that the MAX phases completely converted to MXene phases. Moreover, there are no Al signals in the energy-dispersive X-ray spectroscopies (EDSs) of delaminated Nb_2_C and Ta_2_C MXenes, which also demonstrate the successful synthesis of MXene phases. Raman spectra of MAX phase and MXenes are shown in Fig. S8. The coupling vibration modes of Nb/Ta and C atoms with Al atoms in the wavenumber range of 100–400 cm^−1^ become suppressed or even disappeared after HF etching, which also confirm the complete removal delamination of Al layer in Nb_2_AlC and Ta_2_AlC MAX. X-ray photoelectron spectroscopy (XPS) is used to study the information of surface chemical state. In addition to the inherent Nb-C bond and Ta-C bond, the Nb 3d and Ta 4f XPS spectra of delaminated Nb_2_C and Ta_2_C MXenes (Fig. S7c, f) show the presence of NbC_x_O_y_ and TaC_x_O_y_. The NbC_x_(OH)_y_ and TaC_x_(OH)_y_ are also demonstrated existing in the O 1 s XPS spectra of delaminated Nb_2_C and Ta_2_C MXenes (Fig. S9). It is worthwhile to note that the high oxygen content of Nb_2_C and Ta_2_C MXenes, which is likely originated from the water intercalation between the MXene layers, would be difficult to remove completely. Furthermore, the presence of Nb^2+^ in Nb 3*d* XPS spectra of MeB-Nb_2_C complex and the increase in Ta^4+^ content in Ta 4f XPS spectra of MV-Ta_2_C complex (Fig. S10) both indicate Nb_2_C and Ta_2_C MXenes substrates tend to gain electrons, which demonstrate the interaction between molecules and the substrates.

### SERS Performances of Nb_2_C and Ta_2_C MXenes

**Fig. 4:**
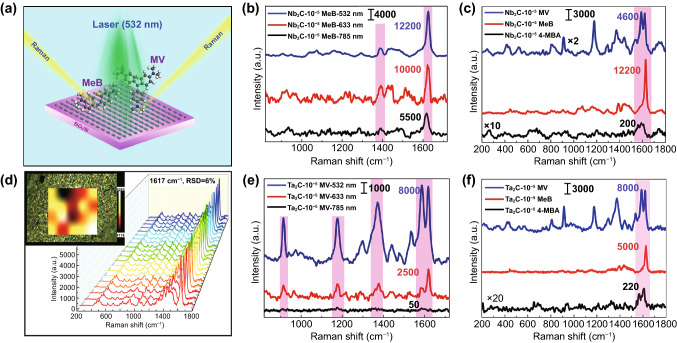
**a** Raman schematic diagram of the Nb_2_C/Ta_2_C NSs as the substrates for the Raman scattering of MeB and MV molecule under the excitation laser of 532 nm. **b**, **e** Raman spectra of 10^−5^ M MeB on Nb_2_C NSs substrates (**b**) and 10^−5^ M MV on Ta_2_C NSs substrates (**e**) under the different wavelength laser excitation of 532 nm, 633 nm, and 785 nm. **c, f** Raman spectra of 10^−5^ M 4-MBA, MeB, MV on Nb_2_C NSs (**c**) and Ta_2_C NSs (**f**) substrates under the excitation laser of 532 nm. **d** The Raman signals of 10^−5^ M MeB collected from 20 selected points on the Nb_2_C NSs substrates and the corresponding Raman mapping image at 1617 cm^−1^ with the area of 40 × 50 μm^2^ (the step size is 10 μm)

The SERS performance of Nb_2_C and Ta_2_C MXenes was researched with the guidance of theoretical calculations. The Raman scattering diagram of probe molecules and substrates is shown in Fig. 4a. Two conventional probe molecules MeB and MV, whose Raman intensity is significantly enhanced according to the calculated static Raman spectra, were selected to detect the SERS sensitivity of Nb_2_C and Ta_2_C MXenes. Firstly, in order to verify the higher SERS effect of MeB on Nb_2_C MXene and MV on Ta_2_C MXene with the irradiation of the optimal resonance excitation wavelength, we investigated the Raman enhanced effect of 10^−5^ M molecules on MXene substrates under the different excitation wavelengths of 532, 633, and 785 nm (Fig. 4b, e). It is found that MeB molecules on Nb_2_C MXene substrates and MV molecules on Ta_2_C MXene substrates have a stronger Raman enhancement under an excitation laser of 532 nm than that of the other two excitation lasers of 633 and 785 nm, which is consistent with the theoretical predictions. Moreover, the more the experimental detecting wavelength deviated from the charge transfer resonance excitation wavelength of 532 nm, the lower the Raman intensity of these complexes. In the Raman spectra of Nb_2_C-MeB complex, Raman vibration modes of the symmetric C-N stretches at 1402 cm^−1^ and the asymmetric stretching vibration of benzene rings at 1620 cm^−1^ are both obviously enhanced. Similarly, the Raman lines of MV molecule at 915, 1176, and 1371, 1617 cm^−1^, which can be assigned to the bending motions of carbon, the asymmetric stretching vibration of benzene ring, the symmetric stretching vibration of benzene ring, respectively, are all greatly enhanced by absorbing on the Ta_2_C MXene substrate. Under the irradiation of the optimal resonance excitation wavelength of 532 nm, the SERS activities of three probe molecules (4-MBA, MeB, MV) with different resonance excitation wavelengths were investigated (Fig. 4c, f). Obviously, Nb_2_C MXene substrate exhibits a stronger SERS enhancement on MeB molecules, while the Raman intensity of the Ta_2_C-MV complex is higher than the other two molecules. This conclusion is consistent with the calculation results of the static Raman spectra. In addition, according to the Raman mapping of 10^−5^ M MeB on the Nb_2_C MXene substrate at 1617 cm^−1^ (Fig. 4d), the relative standard deviation (RSD) is 6.0%, which implies the excellent uniformity of enhanced effect for molecular Raman signal.

**Fig. 5:**
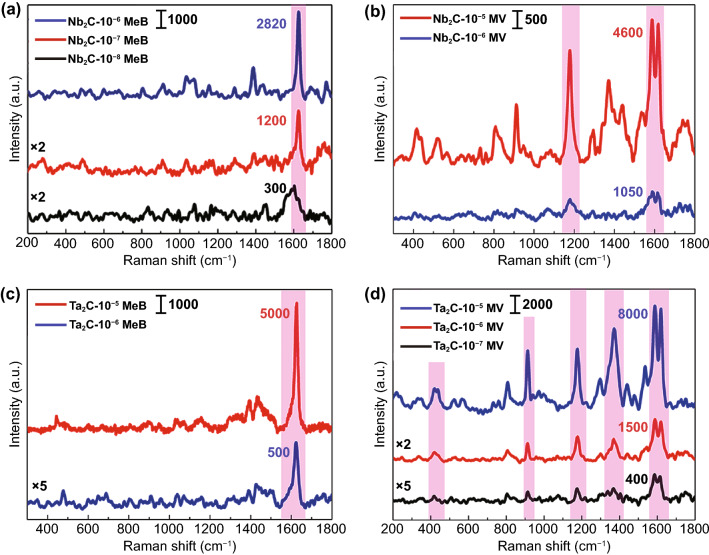
**a** Raman spectra of MeB with three different concentrations, 10^−6^ M, 10^−7^ M and 10^−8^ M on Nb_2_C NSs substrates. **b** Raman spectra of 10^−5^ M and 10^−6^ M MV on Nb_2_C NSs substrates. **c** Raman spectra of 10^−5^ M and 10^−6^ M MeB on Ta_2_C NSs substrates. **d** Raman spectra of MV with three different concentrations of 10^−5^ M, 10^−6^ M and 10^−7^ M on Ta_2_C NSs substrates

In order to explore the SERS sensitivity of MXene substrates, we detected the Raman spectra of probe molecules with different concentrations after being adsorbed to the substrates. With respect to MeB molecules, a weak Raman signal was still detected by adsorbing to Nb_2_C MXene substrates even the molar concentration was diluted to 10^−8^ M (Fig. 5a). When the solution concentration of MV was diluted to 10^−7^ M, greatly enhanced Raman signal could be obtained by adsorbing to Ta_2_C MXene substrates (Fig. 5d). Based on the equation of SERS enhancement factor [35]: ${\text{EF}} = \frac{{I_{\text{SERS}} }}{{I_{\text{prob}} }} \times \frac{{C_{\text{prob}} }}{{C_{\text{SERS}} }}$ (the detailed calculation procedures are shown in the Supporting Information S1), the SERS enhanced factors EFs of 10^−7^ M MeB and 10^−6^ M MV on Nb_2_C MXene substrates at 1617 cm^−1^ with the irradiation laser of 532 nm are 3.0 × 10^6^ and 1.5 × 10^5^ (Fig. 5a, b). By analogy, with regarding to Ta_2_C MXene substrates, the SERS EFs of 10^−6^ M MeB and 10^−7^ M MV are determined to 3.8 × 10^5^ and 1.4 × 10^6^ (Fig. 5c, d). Moreover, a surprising result is that MV on Ta_2_C MXene and MeB on Nb_2_C MXene all possess a low detection limit of 10^−7^ M and 10^−8^ M, which is excellent among the reported pure MXene substrates with the 532 nm laser excitation according to the Table S1 of LODs and EFs on carbide substrates. Furthermore, we also report, for the first time, Nb_2_C and Ta_2_C MXene substrates exhibit an excellent SERS sensitivity.

### SERS Detection of SARS-CoV-2 S Protenin

Based on the excellent SERS sensitivity of Nb_2_C and Ta_2_C MXenes, it can not only be applied to the detection of organic pollutants in the water environment but also be considered for the rapid detection of virus particles. The SARS-CoV-2 with a corolla-like morphology are approximately 100 nm in diameter and larger than molecules that are conventionally analyzable by SERS. The coronavirus is covered by spike (S) glycoprotein with the size of several nanometer, which is a key target for the development of vaccines and therapeutic antibodies, as well as clinical diagnostics [57]. The detectable characteristic Raman signals usually contained the surface S protein will tend to dominate the SERS-Raman spectra of coronavirus. Therefore, the detected results of SARS-CoV-2 S protein can represent the existence of novel coronavirus to a certain extent. Additionally, the SARS-CoV-2 S protein is non-infectious, which can ensure the safety for experimenters. Consequently, we believed that real-time coronavirus detection can be achieved by measuring the Raman signals from the surface molecules of coronavirus.

**Fig. 6:**
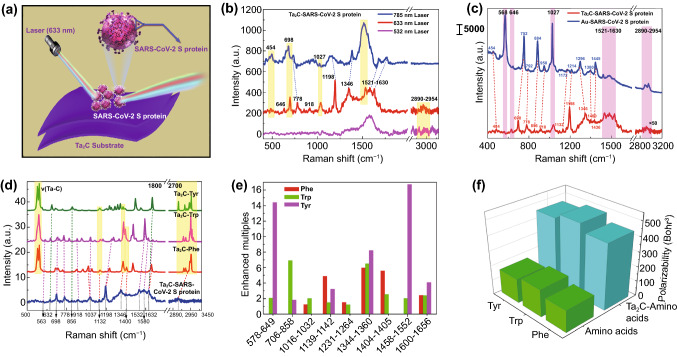
**a** Raman schematic diagram of the Ta_2_C NSs for the Raman scattering of SARS-CoV-2 S protein under the excitation laser of 633 nm. **b** Raman spectra of 10^−9^ M SARS-CoV-2 S protein on Ta_2_C NSs substrates under the different laser wavelength excitation of 532 nm, 633 nm, and 785 nm. **c** Raman spectra of SARS-CoV-2 S protein on Ta_2_C NSs and Au NPS substrates. **d** Static Raman spectra of amino acid molecules of Phe, Trp, Tyr, and their corresponding Ta_2_C-amino acid molecules complexes, as well as the experimental Raman spectra of SARS-CoV-2 S protein on Ta_2_C NSs. **e** Raman enhanced multiples of Ta_2_C-amino acid molecules complexes for nine calculated Raman models. **f** Calculated polarizabilities of amino acid molecules of Phe, Trp, Tyr, and their corresponding Ta_2_C-amino acid molecules complexes

As shown in Fig. 6a, the diluted SARS-CoV-2 S protein molecules were adsorbed on Nb_2_C and Ta_2_C MXenes for Raman detection. The Raman peaks of SARS-CoV-2 S protein obtained under the excitation laser of 633 nm is more obvious and stronger than that of the other two excitation lasers of 533 and 785 nm (Figs. 6b and S11). However, due to the selectivity of SERS enhancement effect of Nb_2_C and Ta_2_C MXenes substrates to molecules, Ta_2_C MXene has a more excellent SERS enhancement on the SARS-CoV-2 S protein. Moreover, its detection limit is as low as 5 × 10^−9^ M, which is beneficial to control the spread of SARS-CoV-2 virus based on SERS technology (the calculation details are shown in the Supporting Information S2) [58–60]. In order to more accurately identify the Raman peaks of SARS-CoV-2 S protein on Ta_2_C MXene substrates, the Raman spectra of SARS-CoV-2 S protein on Au nanoparticle substrate were used as reference (Fig. 6c). Analysis results indicated that the Raman peaks of SARS-CoV-2 S protein on both substrates of Ta_2_C MXene and Au nanoparticles match better with some Raman peaks shift due to the difference in SERS enhancement mechanism and the amount of charge transfer between these two SERS substrates (Table S2). In the Raman spectra of SARS-CoV-2 S protein on Ta_2_C substrates, Raman peaks at 563, 1346, and 1532 cm^−1^ can be assigned to Raman vibration modes of Amide V, Amide III, and Amide II, respectively. Raman lines at 632, 1400, and 1580 cm^−1^ are corresponding to the C–C twisting vibration mode, the N–C stretching mode and the aromatic ring stretching mode of tyrosine (Tyr), respectively. The Raman signal at 856, 698, 1198, and 1400 cm^−1^ are all enhanced, which are assigned to the deformation modes of N–H bonds and C–H bonds, as well as the stretching modes of aromatic ring, C–C_6_H_5_, N–C bond of tryptophan (Trp), respectively. The enhanced Raman peaks at 1037, 1198, 778, and 1580 cm^−1^ are originated from the in-plane deformation mode of C–H bond, the stretching modes of C-C_6_H_5_ and aromatic ring of phenylalanine (Phe), respectively. The Raman shifts at 918, 1132, 1632, and 2890-2950 cm^−1^ are attributed to the stretching modes of C–C bond in skeleton, C–N bond, C=C bond, and C–H bond in aliphatic group of amino acid. Above analysis results on Raman vibration modes indicated that the significantly enhanced Raman peaks of SARS-CoV-2 S protein were mostly attributed to Raman vibration modes of three amino acids of Tyr, Trp, and Phe. In addition, the number of amino acids in the gene sequence of SARS-CoV-2 S protein (Supporting Information S2 and Fig. S12a) showed that three amino acids of Tyr, Trp, and Phe were abundant. Therefore, the static Raman spectra and the polarizability of amino acids and their corresponding Ta_2_C-amino acids complexes were calculated by DFT to verify vibration modes of Raman peaks and analyze the SERS enhancement effect of Ta_2_C MXene on amino acids of Phe, Trp, and Tyr. According to the static Raman spectra (Figs. 6d and S12), Raman vibration modes of Ta_2_C-Phe, Ta_2_C-Trp, Ta_2_C-Tyr complexes can completely match the experimental Raman peaks, which theoretically verified the accuracy of identifying the Raman peaks of SARS-CoV-2 S protein. In addition, the enhancement multiples of static Raman peaks and the polarizability both indicated that Ta_2_C MXene substrate exhibited a significant SERS enhancement effect on amino acids (Fig. 6e, f). This result also indicated that the CM whose charge transfer played a major role has contributed to the SERS detection of SARS-CoV-2 S protein. Additionally, the HOMO–LUMO energy gaps of these three amino acids on Ta_2_C clusters were significantly reduced to 1.93, 1.85, and 1.84 eV (Fig. S13), which indicated that the photo-induced charge transfer resonance between these three amino acids and the Ta_2_C MXene can be better excited by the 633 nm laser. This analysis results also explained an experimental result that the better SERS performance can be excited by 633 nm laser. In a word, the sensitive detection of SARS-CoV-2 S protein and the accurate identification of Raman peaks have been completed by using Ta_2_C MXene substrate, which is of great significance for real-time monitoring and early warning of novel coronavirus based on SERS technology.

## Conclusions

In summary, motivated by the require of virus detection for ultra-sensitivity semiconductor-based SERS substrates, Nb_2_C and Ta_2_C MXenes with remarkable SERS sensitivity were developed, and an effective experimental design was presented to optimize the SERS sensitivity. Based on DFT calculations and FDTD simulations, both Nb_2_C and Ta_2_C MXene substrates exhibit a remarkable SERS enhancement on their befitting molecules MeB and MV, respectively. The excellent SERS performance of Nb_2_C-MeB complex and Ta_2_C-MV complex were synergistically contributed by the PICT resonance enhancement and electromagnetic enhancement, and its optimal resonance excitation wavelengths of 532 nm were determined and demonstrated in experiments. Then, with the guidance of above theoretical predictions, Nb_2_C and Ta_2_C MXenes with excellent SERS sensitivity were firstly reported and the SERS enhancement factors of MeB on Nb_2_C MXene and MV on Ta_2_C MXene were successfully optimized to reach up to 3.0 × 10^6^ and 1.4 × 10^6^, respectively. Especially, Nb_2_C MXene exhibited a low detection limit to 10^−8^ M for MeB molecule, which is excellent among the reported pure MXene substrates with the 532 nm laser excitation. Significantly, SARS-CoV-2 S protein can be sensitively detected and accurately identified by Ta_2_C-based substrates. Moreover, the detection limit of SARS-CoV-2 S protein is as low as 5 × 10^−9^ M, which is of great significance for real-time monitoring and early warning of novel coronavirus based on SERS technology. In conclusion, above research from theoretical calculations to experiments not only give helpful guidance for exploring the other novel SERS-active semiconductor materials but also provide a potential candidate for the practical application of SERS technology.

## Electronic Supplementary Material

Below is the link to the electronic supplementary material.

Supplementary material 1 (PDF 1890 kb)
