# Supplementary material for: Charge-Transfer Resonance and Electromagnetic Enhancement Synergistically Enabling MXenes with Excellent SERS Sensitivity for SARS-CoV-2 S Protein Detection
Source: Nanomicro Lett. 2021 Jan 5;13:52. doi: 10.1007/s40820-020-00565-4 (PMC7783703; doi:10.1007/s40820-020-00565-4)
Supplement: Supplementary file 1 — Supplementary material 1 (PDF 1890 kb) [file 40820_2020_565_MOESM1_ESM.pdf]

Supporting Information for

Charge-Transfer Resonance and Electromagnetic Enhancement Synergistically Enabling MXenes with Excellent SERS Sensitivity for SARS-CoV-2 S Protein Detection

Yusi Peng[1, 2, 3], Chenglong Lin[1, 2, 3], Li Long[4], Tanemura Masaki[5], Mao Tang[1], Lili Yang[1, 2, 3], Jianjun Liu[1], Zhengren Huang[1], Zhiyuan Li[4], Xiaoying Luo[6], John R. Lombardi[7], Yong Yang[1, 3, *]

[1]State Key Laboratory of High-Performance Ceramics and Superfine Microstructures, Shanghai Institute of Ceramics, Chinese Academy of Sciences, 1295 Dingxi Road, Shanghai 200050, P. R. China

[2]Graduate School of the Chinese Academy of Sciences, No.19(A) Yuquan Road, Beijing 100049, P. R. China

[3]Center of Materials Science and Optoelectronics Engineering, University of Chinese Academy of Sciences, Beijing 100049, P. R. China

[4]School of Physics and Optoelectronics, South China University of Technology, Guangzhou 510641, P. R. China

[5]Department of Frontier Materials, Nagoya Institute of Technology, 466-8555, Nagoya, Japan

[6]State Key Laboratory of Oncogenes and Related Genes, Shanghai Cancer Institute, Renji Hospital, Shanghai Jiaotong University School of Medicine, Shanghai 200032, P. R. China

[7]City College, CUNY, New York, NY, USA

*Corresponding author. E-mail: yangyong@mail.sic.ac.cn (Yong Yang)

S1 Supplementary Figures

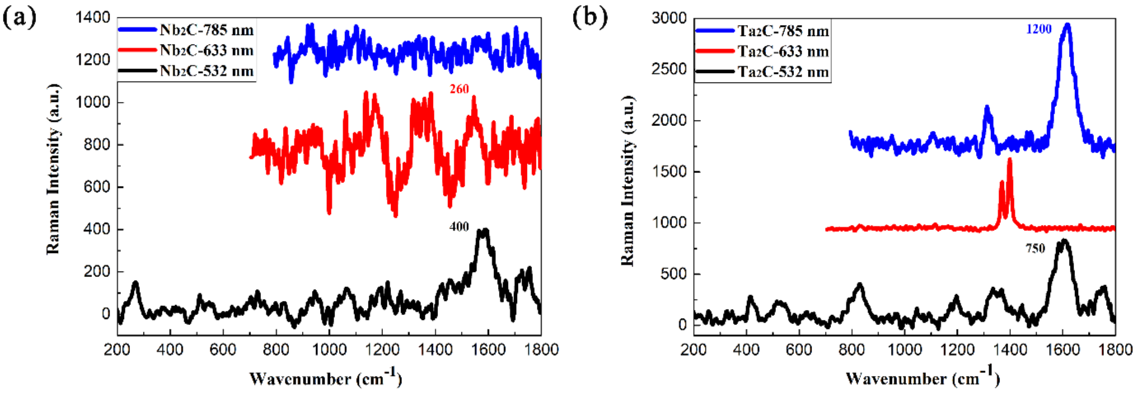

Fig. S1 SERS spectra of Nb_2C (**a**) and Ta_2C (**b**) substrates with different excitation wavelengths of 532 nm, 633 nm, 785 nm

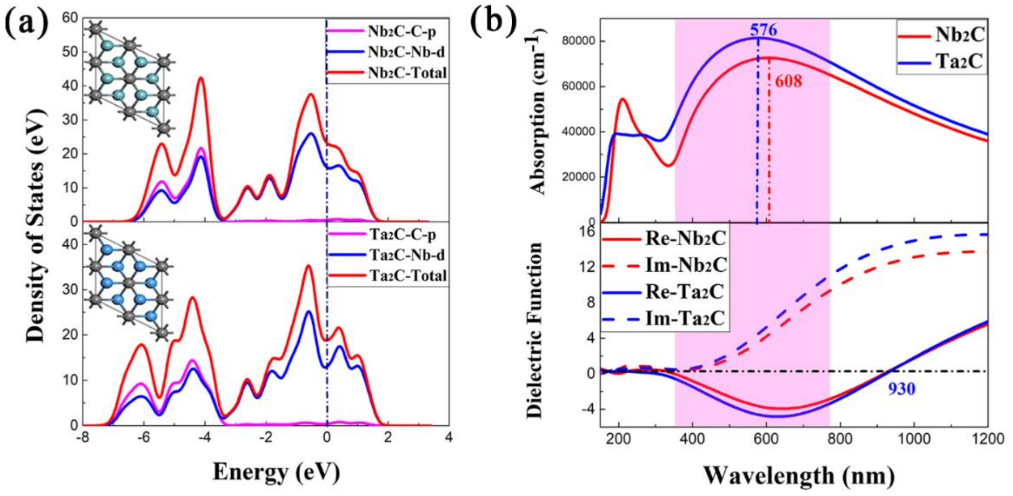

Fig. S2 (**a**) Partial density of states of Nb_2C and Ta_2C bulk structure. (**b**) Calculated absorption spectra and dielectric function spectra of Nb_2C and Ta_2C bulk structure

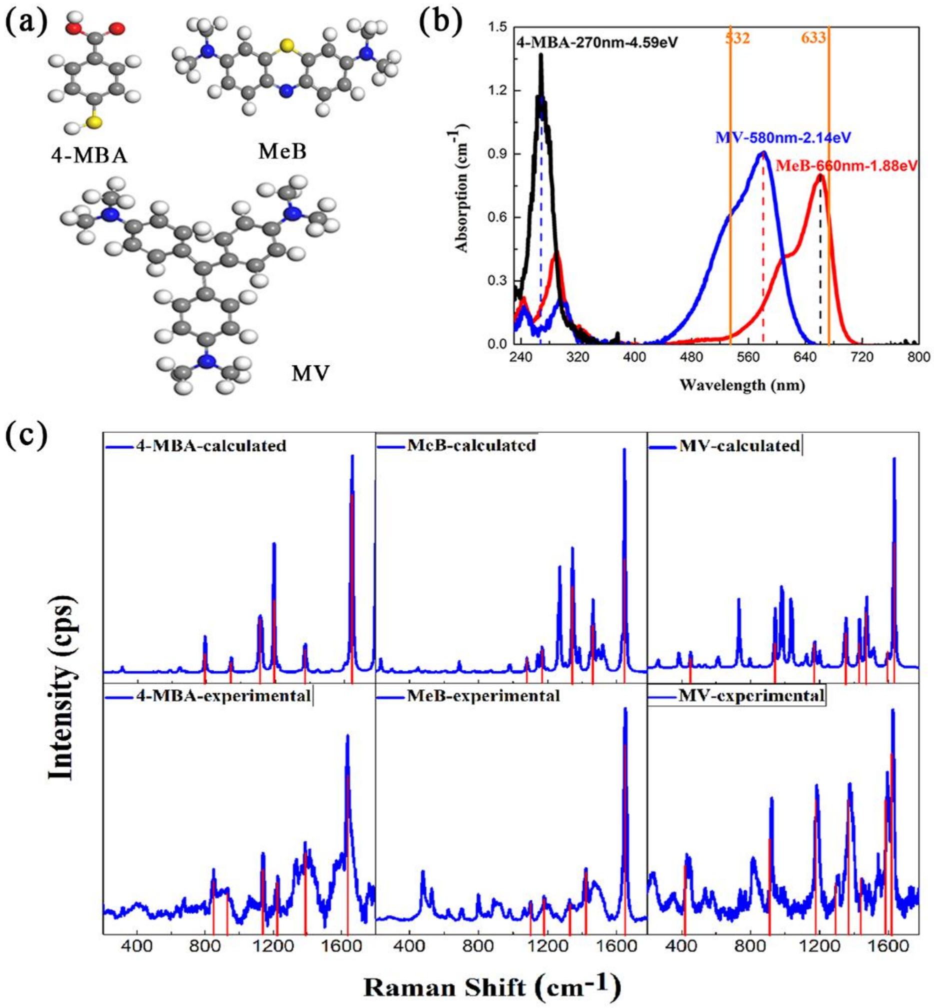

Fig. S3 (**a**) Basic models of the isolated molecules of 4-MBA, MeB, MV. (**b**) Experiment absorption spectra of probe molecules 4-MBA, MeB, MV. (**c**) Calculated and experimental Raman spectra of 4-MBA, MeB, MV

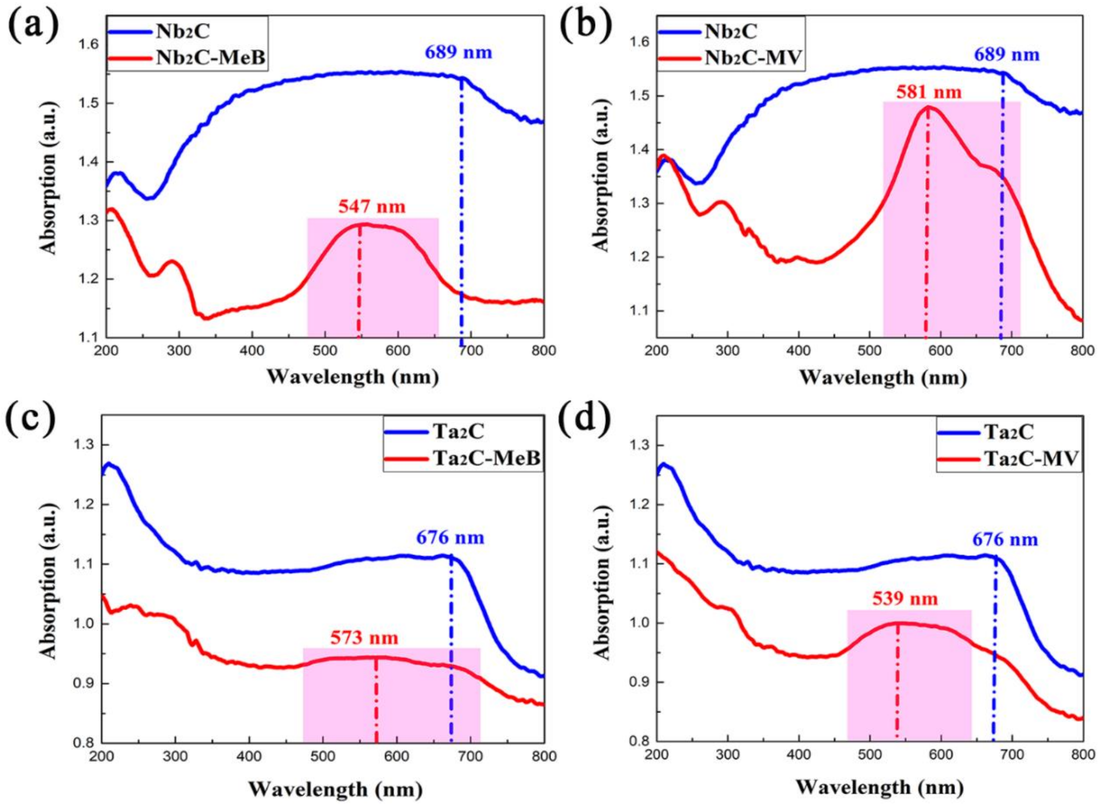

Fig. S4 Experiment ultraviolet-visible absorption spectra of probe molecules MeB-Nb_2C (**a**), MV-Nb_2C (**b**), MeB-Ta_2C (**c**), MV-Ta_2C (**d**)

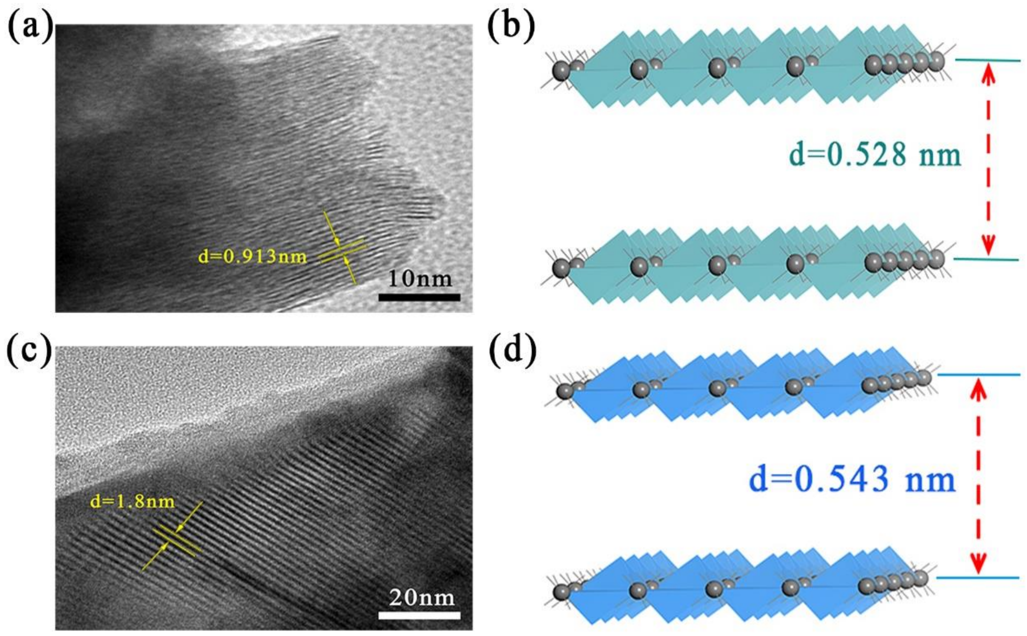

Fig. S5 (**a, c**) TEM images of Nb_2C Mxene (**a**) and Ta_2C Mxene (**c**) before the delamination process. (**b, d**) Schematic diagram of atomic interlayer distance of Nb_2C Mxene (**b**) and Ta_2C Mxene (**d**)

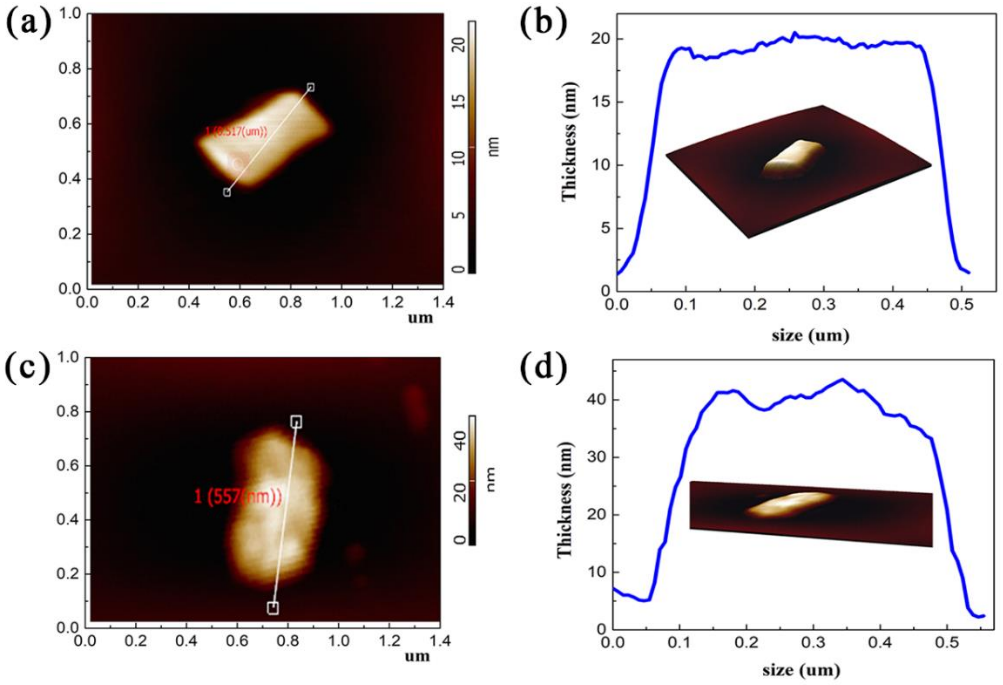

Fig. S6 (**a, c**) AFM images of Nb_2C Mxene (**a**) and Ta_2C Mxene (**c**) after the delamination process. (**b, d**) The thickness and the corresponding 3D AFM images of Nb_2C Mxene (**b**) and Ta_2C Mxene (**d**)

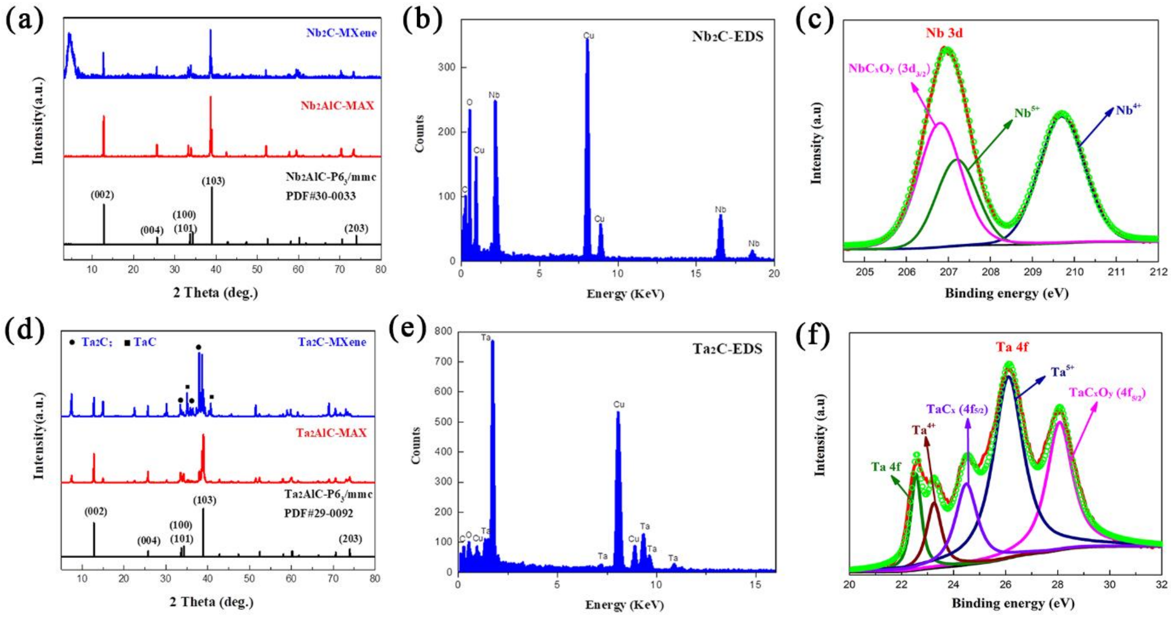

Fig. S7 (**a, d**) XRD patterns of Nb_2AlC ceramics and Nb_2C nanosheets (**a**), Ta_2AlC ceramics and Ta_2C nanosheets (**d**). (**b, e**) EDS spectra of delaminated Nb_2C (**b**) and Ta_2C (**e**) nanosheets. (**c, f**) Nb 3d and Ta 4f XPS spectra of delaminated Nb_2C (**c**) and Ta_2C (**f**)

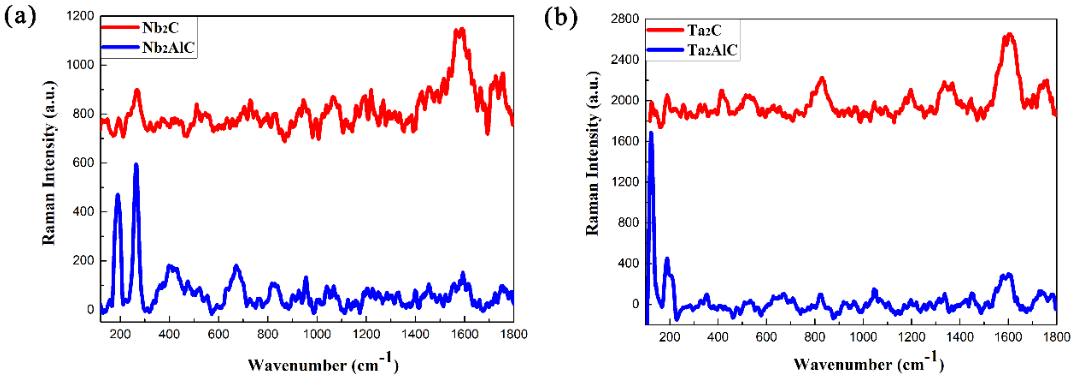

Fig. S8 SERS spectra of Nb_2AlC, Nb_2C substrates (**a**) and Ta_2AlC, Ta_2C substrates (**b**) under the excitation laser of 532 nm

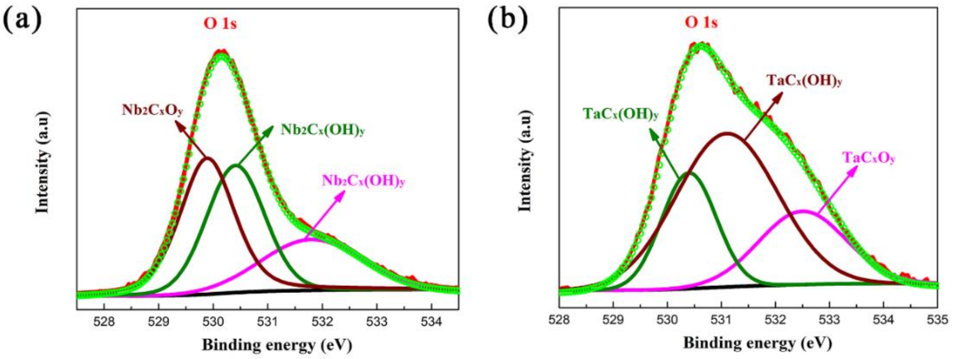

Fig. S9 (**a, b**) O 1s XPS spectra of delaminated Nb_2C (**a**) and Ta_2C (**b**)

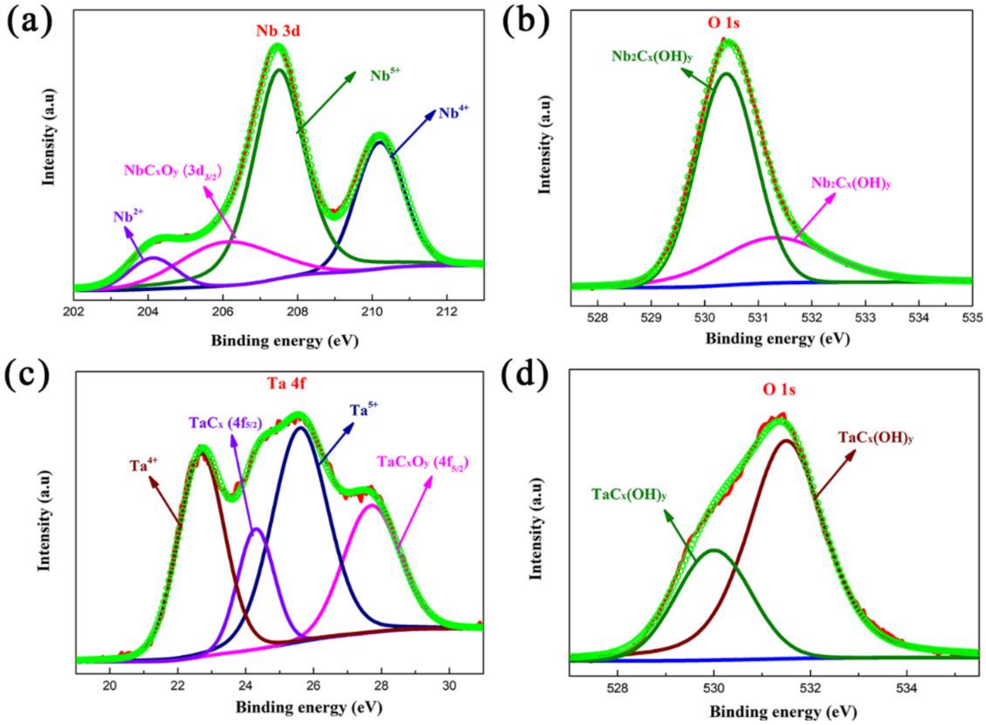

Fig. S10 (**a, c**) Nb 3d and Ta 4f XPS spectra of MeB-Nb_2C complex (**a**) and MV-Ta_2C complex (**c**), respectively. (**b, d**) O 1s XPS spectra of MeB-Nb_2C complex (**b**) and MV-Ta_2C complex (**d**)

S2 Enhancement Factor (EF) Calculations

SERS enhancement factors for Nb_2C and Ta_2C MXenes substrates were calculated by the following general formula [S1]:

$$EF=\frac{I_{SERS}}{I_{prob}}\times\frac{C_{prob}}{C_{SERS}} \quad (S1)$$

Based on formula (S1), I_{SERS} and I_{prob} are the Raman intensity at a selected Raman peak of molecule-semiconductor complex and MV or MeB probe molecules. C_{SERS} is the SERS detected concentration of MV or MeB molecules in the scattering volume. C_{prob} is the pristine concentration of MV or MeB molecules in the scattering volume for Raman detection. In the Raman scattering detection region, the C_{prob} of MV and MeB molecules are 2.718 M and 3.13 M, respectively. Under the excitation of 532 nm laser, the Raman intensity I_{prob} of MV molecule at 1617 cm^{-1} is 477481.4, and I_{prob} of MeB at 1620 cm^{-1} is 335466.26. All Raman spectra complex were detected with the following test conditions: the irradiation power of excitation light is 50 mW×1% of 532 nm, and the light irradiation time of each point is 20 s, as well as the light transmission efficiency of the Raman spectrometer is about 20%.

As for MV molecules with the irradiation of 532 nm laser, $EF=\frac{I_{SERS}}{C_{SERS}}\times 5.69\times 10^{-6}$

As for MeB molecules with the irradiation of 532 nm laser, $EF=\frac{I_{SERS}}{C_{SERS}}\times 9.33\times 10^{-6}$

Therefore, the SERS enahncement factors of Nb_2C and Ta_2C MXenes substrates can be calculated from the measured Raman spectra.

As for 10^{-6} M MV adsorbing on Nb_2C MXene, I_{SERS} = 67177.188 and EF = 3.8×10^5;

As for 10^{-7} M MV adsorbing on Ta_2C MXene, I_{SERS} = 24244.117 and EF = 1.4×10^6;

As for 10^{-7} M MeB adsorbing on Nb_2C MXene, I_{SERS} = 32185.306 and EF = 3.0×10^6;

As for 10^{-6} M MeB adsorbing on Ta_2C MXene, I_{SERS} = 15899.077 and EF = 1.5×10^5;

Based on above calculations of our works, SERS enhancement factors and the corresponding detection limits of all reported MXene carbide materials with SERS activity were summarized in the Table S1.

Table S1 Reported enhancement factors on different semiconductor nanostructure substrates

**SERS substrates**	**Probe molecules**	**Excitation wavelength (nm)**	**EF**	**Detection limits**
Ag@ Ti_3C_2 MXenes,<br>Au@ Ti_3C_2 MXenes,<br>Pd @ Ti_3C_2 MXenes [2]	MB	632.8	1.5×10^5<br>1.17×10^5<br>9.61×10^4	10^{-6} M
The films of Ti_3C_2 MXenes [1]	R6G	488	1.2×10^6	
	MB	488	1.6×10^6	10^{-7} M
Multi-layers of Ti_3C_2 MXene-SO_3H [3]	R6G	532	2×10^5	
Monolayer of Ti_3C_2 MXene [4]	R6G	532	3.82×10^8	10^{-11} M
Ti_3C_2 MXenes (previous work)	MV	532	2.84×10^5	10^{-6} M
	MeB	785	3.24×10^6	10^{-7} M
Ti_2C MXenes (previous work)	MV	532	4.15×10^4	10^{-6} M
**Nb_2C MXenes (this work)**	MV	532	3.8×10^5	10^{-6} M
	**MeB**	**532**	**3.0×10^6**	**10^{-8} M**
**Ta_2C MXenes (this work)**	**MV**	**532**	**1.4×10^6**	**10^{-7} M**
	MeB	532	1.5×10^5	10-6 M

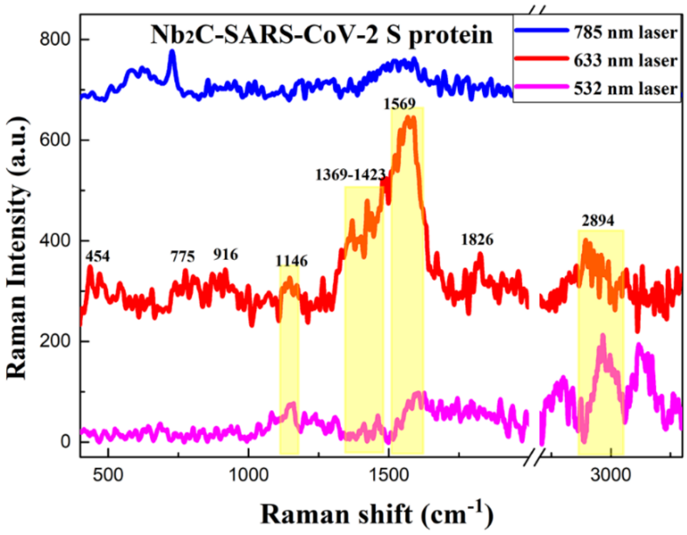

Fig. S11 Raman spectra of 5×10^{-9} M SARS-CoV-2 S protein on Nb_2C NSs substrates under the different wavelength excitation lasers of 532 nm, 633 nm, 785 nm

S3 SERS Detection of SARS-CoV-2 S Protein

S3.1 Gene sequence of SARS-CoV-2 S protein

QCVNLTTRTQLPPAYTNSFTRGVYYPDKVFRSSVLHSTQDLFLPFFSNVTWFHAIHVSGTNG
TKRFDNPVLPFNDGVYFASTEKSNIIRGWIFGTTLDSKTQSLLIVNNATNVVIKVCEFQFCND
PFLGVYYHKNNKSWMESEFRVYSSANNCTFEYVSQPFLMDLEGKQGNFKNLREFVFKNID
GYFKIYSKHTPINLVRDLPQGFSALEPLVDLPIGINITRFQTLLALHRSYLTPGDSSSGWTAGA

AAYYVGYLQPRTFLLKYNENGTITDAVDCALDPLSETKCTLKSFTVEKGIYQTSNFRVQPTE
SIVRFPNITNLCPFGEVFNATRFASVYAWNRKRISNCVADYSVLYNSASFSTFKCYGVSPTKL
NDLCFTNVYADSFVIRGDEVRQIAPGQTGKIADYNYKLPDDFTGCVIAWNSNNLDSKVGGN
YNYLYRLFRKSNLKPFERDISTEIYQAGSTPCNGVEGFNCYFPLQSYGFQPTNGVGYQPYRV
VVLSFELLHAPATVCGPKKSTNLVKNKCVNFNFNGLTGTGVLTESNKKFLPFQQFGRDIADT
TDAVRDPQTLEILDITPCSFGGVSVITPGTNTSNQVAVLYQDVNCTEVPVAIHADQLTPTWR
VYSTGSNVFQTRAGCLIGAEHVNNSYECDIPIGAGICASYQTQTNSPRRAR

S3.2 Detection limits of SARS-CoV-2 S protein

The SARS-CoV-2 S protein solution was purchased from Sanyou Biopharmaceuticals (2.38 mg/mL, 20 μg/tube, Shanghai, China) and diluted to 1.5 mL by adding PBS solution. Then, the 40 μL of diluted SARS-CoV-2 S protein solution was added to 1.5 mL PBS solution, and the 0.02 g of Nb_2C and Ta_2C Mxene powder was immersed in it for 30 min. As for the purchased SARS-CoV-2 S protein solution, the number of mole (n) and molecules (N) are shown below:

$$n=m/M=20\times10^{-6}\ g/75243=2.66\times10^{-10}\ mol$$

$$N=n\times N_A=2.66\times10^{-10}\times 6.02\times10^{23}=1.6\times10^{14}$$

As for the diluted SARS-CoV-2 S protein solution, the number of mole (n_1) and molecules (N_1) are shown below:

$$N_1=1.6\times10^{14}\times40/1500=4.26\times10^{12}$$

$$n_1=N_1/N_A=4.26\times10^{12}/6.02\times10^{23}=7.08\times10^{-12}\ mol$$

Based on the results of Raman tests, the detection limit of Ta_2C Mxene substrate for SARS-CoV-2 S protein is shown below:

$$LOD=n_1/V_1=7.08\times10^{-12}/1.5\times10^{-3}=4.72\times10^{-9}\ M\approx 5\times10^{-9}\ M$$

Table S2 Raman models of SARS-CoV-2 S protein in literature, experimental results of molecules on Ta_2C NSs and Au NPS substrates, calculation results of Ta_2C-amino acid molecules complexes

Assignments (Literature)	Reference		Experiment	Calculation	Assignments (Calculation results)
	Literature (cm^{-1})	Au (cm^{-1})	Ta_2C (cm^{-1})	Ta_2C (cm^{-1})	
Amide V	567 [S6]	568	563	578	Trp, v(C-N) in indole ring
Tyr, ρ_τ(C-C)	645 [S5]	646	632	648	Tyr, ρ_τ(C-C)
Trp, v(ϕ)	758 [S5]	752	698	706	Trp, v(ϕ)
Phe, v(ϕ)	778 [S5]	792	778	771	Phe, v(ϕ, indole ring)
Trp, W17 [δ(CH)$_{\phi Ph}$ & δ(N-H)]	880 [S6] 874 [S7]	884	856	858	Tyr & Trp, v(N-H)
Skeleton, v(C-C, α-Helix)	Ref. [S7]	950	918	906	Trp, Skeleton, v(C-C)
Phe, ρ_{ipb}(C-H)	1034 [S8]	1027	1037	1016/1039	Phe & Trp, ρ_{ipb}(C-H) in aromatic ring
v(C-N)	1155 [S5]	1172	1132	1139/1142	v(C-N)
Trp & Phe, v(C-C_6H_5)	1209 [S5]	1214	1198	1231/1254	Phe & Trp, v(C-C_6H_5)
Amide III (α-Helix)	Ref. [S8]	1296	1346	1366/1351 /1372/1344	Skeleton, v(C-C)
Trp, W7 [v(N_1–C_8)]	1365 [S6]	1380	1400	1405/1404	Phe & Trp, v(C-C), v(C-N)
Amide II	1509-1552 1529 [S8]	1521	1532	1552-1590	v(C-C=O)
Tyr & Phe, v(ϕ)	1591 [S8]	1592	1580	1592-1599	v(ϕ) or v(indole ring)
v(C=C)	1617-1680 [S5]	1630	1632	1656/1667 /1665	Phe & Trp & Tyr, v(ϕ) & δ(ϕ)
CH、CH_2 & CH_3 in aliphatic group, v(C-H)	2890-2980 [S5]	2890,2950	2890,2950	3040-3210	Skeleton, v(C-H) & v(N-H)

Abbreviation: δ, deformation; v, stretching; ρ_τ, *twisting vibrations;* ϕ, *aromatic ring;* ρ_{ipb}, *in-plane deformation;*

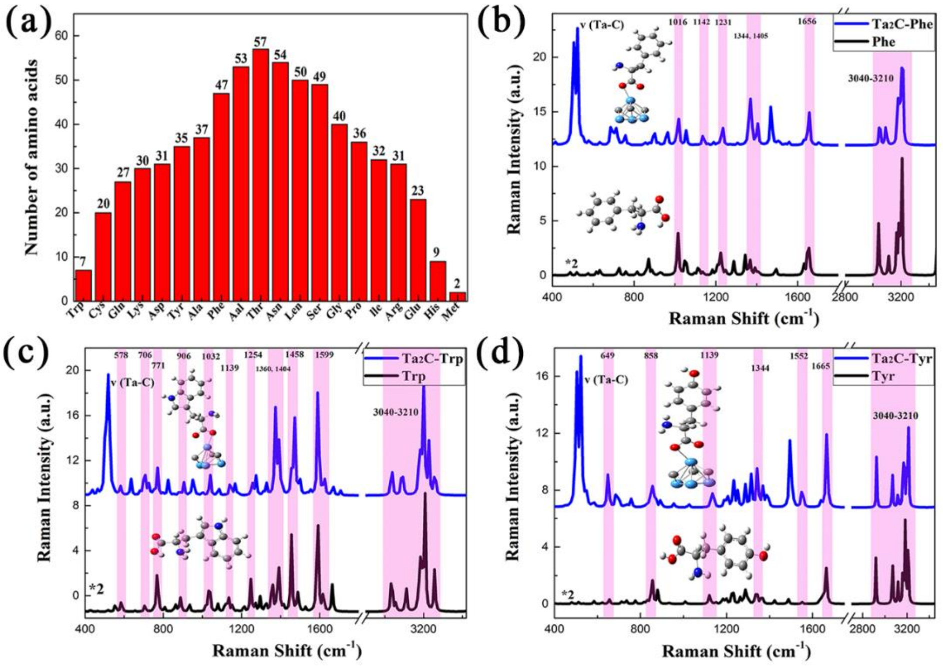

Fig. S12 (**a**) Number of amino acids in the gene sequence of SARS-CoV-2 S protein. (**b-d**) Static Raman spectra of amino acid molecules of Phe and Ta_2C-Phe complex (**b**), Trp and Ta_2C-Trp complex (**c**), Tyr and Ta_2C-Tyr complex (**d**)

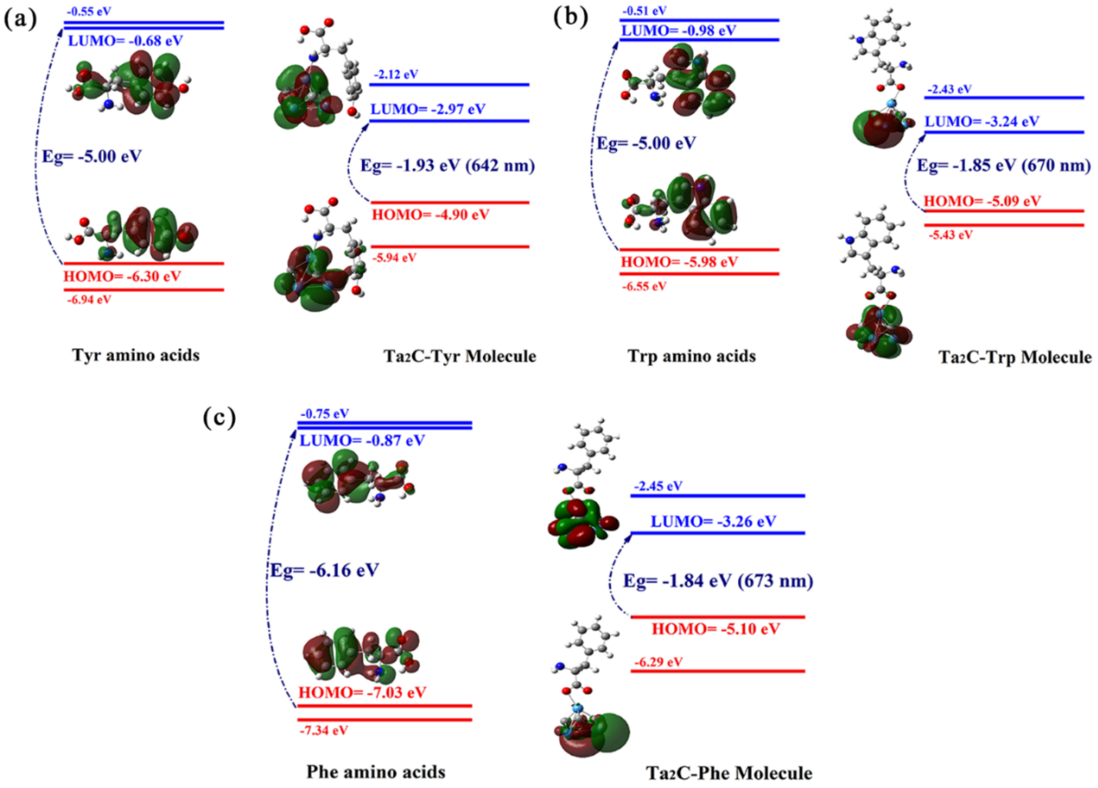

Fig. S13 Energy level distributions and HOMO/LUMO illustrations of Tyr (**a**), Trp (**b**), Phe (**c**) amino acids, and Ta_2C-amino acids complexes obtained by the accurate higher precision DFT calculation

Supplementary References

[S1] A. Sarycheva, T. Makaryan, K. Maleski, E. Satheeshkumar, Y. Gogotsi et al., Two-dimensional titanium carbide (MXene) as surface-enhanced raman scattering substrate. J. Phys. Chem. C **121,** 19983-19988 (2017). https://doi.org/10.1021/acs.jpcc.7b08180

[S2] E. Satheeshkumar, T. Makaryan, M. Yoshimura, One-step solution processing of Ag, Au and Pd@MXene hybrids for SERS. Sci. Rep. **6,** 32049 (2016). https://doi.org/10.1038/srep32049

[S3] K.Y. Chen, X. Yan, J. K. Li, T.F. Jiao, Q.M. Peng et al., Preparation of Self-Assembled Composite Films Constructed by Chemically-Modified MXene and dyes with surface-enhanced Raman scattering characterization. Nanomaterials **9,** 284 (2019). https://doi.org/10.3390/nano9020284

[S4] Y.T. Ye, W.C. Yi, W. Liu, Y. Zhou, H. Bai et al., Remarkable surface-enhanced Raman scattering of highly crystalline monolayer Ti_3C_2 nanosheets. Sci. China Mater. **63(5),** 794–805 (2020). https://doi.org/10.1007/s40843-020-1283-8

[S5] M. Keshavarz, B. Tang, K. Venkatakrishnan, Label-free SERS quantum semiconductor probe for molecular-level and in vitro cellular detection: a noble-metal-free methodology. ACS Appl. Mater. Interfaces **10,** 34886−34904 (2018). https://doi.org/10.1021/acsami.8b10590

[S6] K.L. Aubrey, G.J. Thomas, Raman spectroscopy of filamentous bacteriophage Ff (fd, M13, f1) incorporating specifically-deuterated alanine and tryptophan side chains. Assignments and structural interpretation. Biophys. J. **60(6),** 1337-1349 (1992). https://doi.org/10.1016/S0006-3495(91)82171-3

[S7] S. Krimm, J. Bandekar, Vibrational spectroscopy and conformation of peptides, polypeptides, and proteins. Adv. Protein Chem. **38(C),** 181-364 (1986). https://doi.org/10.1016/S0065-3233(08)60528-8

[S8] J. Bandekar, Amide modes and protein conformation. Biochim. Biophys. Acta **1120(2),** 123-143 (1992). https://doi.org/10.1016/0167-4838(92)90261-B
